# Early detection of learning difficulties using the BADyG-E2r Battery during primary education

**DOI:** 10.1186/s41155-020-00143-y

**Published:** 2020-05-07

**Authors:** Ignasi Navarro Soria, José Manuel García Fernández, Cándido J. Inglés Saura, Marta Real Fernández

**Affiliations:** 1 Department of Developmental Psychology and Didactics, University of Alicante, Alicante, Spain; 2 Department of Health Psychology, Miguel Hernandez University of Elche, Elche, Spain

**Keywords:** Cognitive skills, Mathematics, Language, Learning difficulties, Primary education

## Abstract

The aim of the present study was to assess the predictive capacity of several of the most relevant cognitive skills in the academic field that were evaluated using Differential and General Skills Battery(BADyG-E2r). Particular attention was focused on the variables that need to be overcome regarding the curricular objectives related to pass/fail grading as evaluated by the teachers in the instrumental disciplines of Mathematics and Language. The psychometric battery was applied to the 3rd year students in primary education (a total of 512 students) at 4 public schools that were randomly selected in the province of Alicante (Spain). A follow-up of their academic evolution was under taken until the end of primary education. The obtained results show that high scores in Verbal Reasoning, Numerical Reasoning, and Verbal Syllogisms positively and significantly predict academic success at the end of primary education in the subjects of Language and Mathematics.

## Introduction

Throughout primary education, some students have difficulty achieving the curricular objectives which are expected during their academic year. If difficulties which are initially mild are left unattended, then in many cases they can lead to bigger problems, such as low academic performance and early abandonment of the education system (Duncan et al., 2007; Kern & Friedman, 2009). To a large extent, these latter examples would lead to school failure. Academic difficulties are preceded by weakness in aptitude concerning cognitive processes because such processes allow the student to learn at a pace which is assumed to be reasonable for their age (Grañeras, & Díaz-Caneja,, & Gil, N., 2011; Navarro-Soria, 2016; Nisbett et al., 2012). Therefore, a special focus is required concerning classroom activities with respect to the acquisition of formal knowledge and the development and maturity of cognitive skills, which will allow an adequate access to the curriculum objectives for students.

According to research in the peer-reviewed literature on this subject, it is clear that intelligence and the differential abilities that compose intelligence are the most influential variables when predicting academic success (Fergusson, Horwood, & Ridder, 2005; Gagné & Père, 2001; Kuncel, Hezlett, & Ones, 2004; Kytala & Lehto, 2008; Sternberg & Kaufman, 1998; Strenze, 2007; Taub, Keith, Floyd, & Mcgrew, 2008). Further, it has been verified by research that an optimal cognitive development in certain school aptitudes can be an adequate predictor of academic success for instrumental disciplines such as Mathematics and Language. Thus, verbal skills are considered the best predictor of general academic performance, followed by numerical skills, numerical reasoning, and spatial skills (Burnet & Lane, 1980; Cassidy, Roche, Colbert, Stewart, & Grey, 2016; Cooper & Reagan, 1982; Hawes, Moss, Caswell, & Poliszczuk, 2015; Prior, Bavin, & Ong, 2010; Smith, 1964). This skill hierarchy is modified in the event of predicting performance for specific academic areas, such as Mathematics. In this case, numerical skills occupy position one on the ranking, displacing verbal aptitudes to position two. Similarly, if the academic sphere is Language, then numerical aptitude is displaced to position three, yielding verbal aptitudes and logical reasoning in positions one and two, respectively (Cerda et al., 2015; Marrero & Espino, 1988; Spinath, Spinath, Harlaar, & Plomin, 2006; Toll & Van Luit, 2014). In general, such investigations conclude that an important part of learning difficulties in the academic context find their etiology in the cognitive aptitudes which are required for the completion of traditional school activities. Hence, these aptitude deficiencies are the prelude to poor school performance, being detected later on and once the student already presents difficulties in achieving the curricular objectives of his/her academic year (Bennett, Seashore, & Wesman, 1997; Navarro-Soria, 2016; Pérez, Cupani, & Ayllón, 2005; Robles & Vázquez, 2014). As the student gets promoted to higher classes, the curriculum demands increase, and the late detection and initiation of resources for school reinforcement aggravates the difficulties and increases the differences in levels of development in relation to the other students in the class (Navarro-Soria, 2016).

The emphasis of recent research in the literature has been on finding causal relationships between academic performance and the different cognitive skills that allow students to access knowledge in school (Alloway & Passolunghi, 2011; Cornu, Schiltz, Pazouki, & Martin, 2017; Geary & VanMarie, 2016; Harvey & Miller, 2016; Matthews, Lewis, & Hubbard, 2015; Navarro-Soria & González-Gómez, 2010; Pitchford, Papini, Outhwaite, & Gulliford, 2016; Ye et al., 2016). Based on these causal relationships, programs have been developed to control, manipulate, and modify the maturation and capacity of these cognitive skills, in order to improve the preparation of students so that they can confront the different academic challenges they face during their training (Cassidy et al., 2016; Cheng & Mix, 2014; Di Lieto et al., 2017; Hill, Serpell, & Faison, 2016; Park, Bermudez, Roberts, & Brannon, 2016; Peng & Fuchs, 2016). At the same time, knowing which cognitive skills have a greater influence on academic success and which tools measure these cognitive skills effectively can facilitate an individualized intervention and the implementation of the psychopedagogical resources that currently act on the problem from a scholarly assistance perspective (Rogowsky, Papamichalis, Villa, Heim, & Tallal, 2013), rather than from a preventive perspective (Bergman et al., 2011; Blair, McKinnon, & the Family Life Project Investigators, 2016; Blair & Raver, 2014; Di Lieto et al., 2017; Raver et al., 2011).

As it has been shown, the tests used to measure different cognitive variables are different. An example of this is the Differential Aptitude Test (DAT) (Bennett et l., 1997), the Multiple Intelligences Self-efficacy Inventory Revised (MISEI-R) (Pérez et al., 2005), or the AC-MT test (Alloway & Passolunghi, 2011). The BADyG test, in addition to demonstrating being a more complete measurement covering different fields, has also proved to be a good predictor of academic performance. Authors such as Miñano and Castejón (Miñano, Cantero, & Castejón, 2008; Miñano & Castejón, 2008) found that the variable of Verbal Aptitudes and General Intelligence have a high predictive power for academic performance with its effectiveness being proven not only in schools but also at university level (Vélez-van Meerbeke & Roa-González, 2005).

Taking into account the previously described research results, the focus of the above studies was on explaining what skills justify the difficulties related to mastering the content at middle and upper academic levels. Such learning difficulties are evident, and the first symptoms of potential failure in school have already appeared. We consider it more interesting and novel to focus on the preventive purpose. We can find in scientific literature investigations in this scope in the early educational stages. However, none of these studies uses a longitudinal approach, which would allow to measure students’ cognitive aptitudes at early stages of schooling and therefore predict future learning difficulties, with students who have lower scores in cognitive aptitudes presenting more severe learning difficulties at later stages of education.

The aim of the present study was to assess the predictive capacity of several of the most relevant cognitive skills in the academic field that were evaluated using the Differential and General Skills Battery (BADyG-E2r). The specific objectives of the present study and our research hypotheses are shown in Table 1.

**Table 1:** Objectives and hypotheses of the study

	Objectives	Hypotheses (H)
a	Specify which skills have a greater predictive capacity of a student not repeating grades at the end of primary education	They will be verbal aptitudes (measured by the tests of Verbal Syllogisms and Verbal Reasoning)
b	Assess whether vocabulary knowledge (Verbal Syllogisms) and the understanding of its different uses (Verbal Reasoning) present a predictive power of academic success for both the instrumental subjects (Language and Mathematics)	They will be the same aptitudes in Verbal Syllogisms and Verbal Reasoning that determine the correct differently dimensioned Language usage
c	Determine whether the capacity for carrying out Numerical Calculations and Numerical Problem Solving presents a predictive capacity of academic success in the instrumental subjects	It will be decisive when explaining academic success in the instrumental subject of Mathematics
d	Verify whether the skills in reading and writing, spatial orientation (Spatial Syllogisms), and the understanding and management of planar space (Spatial Reasoning) presents a predictive power of academic success in the instrumental subjects	They will not present a predictive power
e	Determine whether General Intelligence presents a predictive power of academic success in the instrumental subjects	GI will not be the most powerful aptitudes predicting that academic success

## Method

### Participants

For the present study, a random or probability sampling using the sampling unit known as the conglomerate was carried out. The target areas consisted of four public schools in the province of Alicante (Spain), with one school from each cardinal point (north, south, east, and west) of the province with a total of 24 groups/classrooms, and the total number of students evaluated was 607. Information was collected during three school years and from all of the students in the sample who were enrolled in the 3rd year of primary education (8–9 years old).

Hence, the first inclusion criterion was that the subject was enrolled at that moment of time, in the 3rd year of primary education, and that they remained in the center until the end of the said educational stage.

A total number of 22 students (3.6%) were excluded at the end of the follow-up, being those students who did not show continuity in their schooling in the referenced educational centers and for whom their academic evolution could not be followed up. Another inclusion criterion meant that in presenting a General Intelligence higher than 80 measured by WISC-IV since, in the case that it was inferior, the results would be affected by the cognitive capacity of the subject.

The number of excluded students increased to 33 (5.4%). Finally, a total of 40 (6.6%) students were excluded from the sample, being those students who had stopped attending Language or Mathematics classes in the 2nd year of primary education. After the follow-up, the total sample had been reduced to 512 students, and of the students that made up the final sample, 232 were males (45.4%) and 280 (54.6%) were females.

The socioeconomic level of the families was distributed by income between upper-middle 92 (18%), middle 220 (43%), middle-low 122 (24%), and low 78 (15%). With respect to the academic training of the families of the students that made up the sample, 113 (22%) had university studies, 144 (28%) had chosen a vocational training, 209 (41%) had finished secondary school, and 46 (9%) presented studies or qualified training. Regarding the ethnic and cultural origin of the families, 342 (67%) of the sample had families in Spain, 72 (14%) were from Latin America, 41 (8%) were from the Maghreb in North Africa, 31(6%) were from sub-Saharan Africa, and 26(5%) were from other European countries.

Before initiating student evaluation and monitoring in this research, the procedure was presented before the District Education Board which is the highest decision-making body of an educational center in Spain. The District Education Board related to the four target schools from where the sample was to be drawn approved the research procedure for those four schools. In addition, the psychometric evaluation and the corresponding analysis of the results of the evaluation were reported to all families by way of traditional mailing. In the letter that the families received, there was an opportunity for the children to not participate in the investigation. None of the families in the total sample exempted their children from participating in the evaluation and subsequent follow-up. This research was conducted in accordance with the 1964 Declaration of Helsinki and its later amendments. Finally, approval was requested from the Ethics Committee of the University of Alicante, which provides and approves the methodology used, and the approval was assigned the file number UA-2018-03-08.

### Procedure and materials

In order to verify which cognitive skills are the most influential in academic development in primary education, an aptitude assessment was carried out at the beginning of the 3rd year of primary education (8 years of age), through psychometric testing using the collective application of the Battery of Differential and General Abilities BADyG-E2r (Yuste-Hernanz, 2012). Furthermore, at the end of this educational stage, information was collected concerning the academic performance in the subjects of Mathematics and Language and evaluated/measured using the grades that were issued by the center.

As aforementioned, the BADyG-E2r battery measures cognitive development in skills related to verbal, numerical, spatial, and logical reasoning, as well as the ability to solve Verbal Syllogisms, Numerical Syllogisms, and Spatial Syllogisms, and the speed and efficiency with which students solve the academic problems they encounter. The aim was to provide basic information to the teachers concerning the skill levels of the class which permits the adaptation of teaching and learning rhythms to the abilities and real needs of the students (Navarro-Soria & Gonzalez-Gómez, 2010) Table 2.

**Table 2:** BADyG-E2r scales

Variable	Measure
Verbal Reasoning	Ability to understand and express ideas with words. Verbal contents and concepts are used, and recognition of analogy relationships between pairs s asked.
Numerical Reasoning	Problems with change, combination/comparison, and equalization
Spatial Reasoning	Ability to imagine and conceive objects in two or three dimensions Two types of stimuli: firstly with more concrete and perceptible relationships, secondly induction of more abstract relationships between the figures
Logical Reasoning	Ability to solve logical problems, to understand, and to plan
Verbal Syllogisms	Ability to use language (complete meanings or “cloze” test)
Numerical Syllogisms	Ability to manage numbers and quantitative concepts (addition and subtraction)
Spatial Syllogisms	Several mental operations to be performed: shape rotations, comparison of sizes, direction, position, and form
Attention	Ability to quickly discriminate visual differences

The General Intelligence Index (GI) is obtained through the sum of the scores of all the tests. The Intellectual Coefficient (IC) is measured from the direct score in GI and taking into account the chronological age of the student (according to the corresponding age scale). In the exploratory factor analysis, a one-dimensional structure is obtained, which confirms the existence of the said General Intelligence factor.

The scales in Verbal Reasoning, Numerical Reasoning, Spatial Reasoning, and Logical Reasoning each consist of 24 elements that are ordered according to the difficulty index (the first items being solved more than the last items) with five response alternatives. Through verbal analogies, numerical problems, puzzles, relationships between figures, and logical problems, the level of development in the different types of reasoning is measured. However, the Verbal Syllogisms, Numerical Syllogisms, Spatial Syllogisms, and Attention Syllogisms are composed of cloze-type exercises with a range of meanings (multiple*-*choice items) whereby numerical calculations are made, and there are figures in which the student must discriminate between shape rotations, sizes, orientation, or form, and the speed of discrimination of visual differences is documented. Each of the dimensions that are measured by the test is evaluated with a specific response scale, with a time limit for completion of each scale.

This test has been subjected to multiple psychometric controls (Yuste-Hernanz, 2002; Yuste-Hernanz, 2012). In each of these controls, the exercises have been restructured according to the analysis of the performed elements, obtaining information for the sample from which the scale of the test is developed, with the final results pertaining to the Cronbach’s coefficient alpha value of .83 for Verbal Reasoning, .91 for Numerical Reasoning, .79 for Spatial Reasoning, .93 for Logical Reasoning, .77 for Verbal Syllogisms, .85 for Numerical Syllogisms, .83 for Spatial Syllogisms, and .95 for General Intelligence. In turn, in relation to the sample of this study, the Cronbach’s coefficient alpha values have ranged between .76 and .94 for the separate elements and the total had a Cronbach’s coefficient alpha value of .82.

Also, the construct validity of this test was based on its structure in the factorialist and hierarchical psychometric theories that, starting from authors such as Spearman, Cattell, Horn, Eysenk, or Sternberg, nowadays form a solid theoretical line of reflection. Hierarchical models provide a good conceptualization of the aptitudes measured by test and are what best explain the empirical data obtained when solving test elements.

In addition, the academic performance of the students was measured using the grading by the teachers in the subjects of Mathematics and Language during the final year of primary education. Taking into account the cognitive abilities (high, medium, or low) of the students and, based on their school results (pass or fail), the influence of the differences in aptitude on the academic performance has been determined.

### Statistical analysis

Simple logistic regression—the logit model—was used to analyze the influence of the linear combination of the predictor variables, following the forward step-wise selection procedure based on the Wald statistic (García, Alvarado, & Jiménez, 2000). Logistic modeling allows the estimation of the probability of an occurrence, event, or outcome, as opposed to a non-occurrence, in the presence of one or more predictors. This probability is estimated using the odds ratio (OR) statistic. If OR > 1, for each time the event occurs in the absence of the independent variable, it will be given twice if that independent variable is present. However, if OR < 1, then the probability of the event occurring in the absence of the independent variable will be greater than if that independent variable would be present (De Maris, 2003). To analyze the adjustment of the proposed models, two indicators were taken into account: (a) Nagelkerke’s *R*^2^, which indicates the percentage of variance explained by the model (Nagelkerke, 1991), and (b) the percentage of correctly classified cases, which allows to determine to what extent the predictor variable is useful for estimating the criterion variable in the proposed model.

## Results

The data permitted the creation of logistic regression models that make it possible to carry out correct estimations regarding the probability of not repeating a course and to overcome the two instrumental subjects (Language and Mathematics), based on the scores in cognitive maturity in the different cognitive skills that were evaluated.

Table 3 shows the steps followed by the model in the introduction of explanatory variables that have been significant regarding the probability of not repeating a course. For the evaluation in the 3rd year of primary education using the BADYG-E2r, the proposed model allows a correct estimation of 88.8% of cases in Verbal Reasoning, 90.2% in Numerical Reasoning, 88.0% in Spatial Reasoning, 88.4% in Verbal Syllogisms, 87.8% in Numerical Syllogisms, 87.4% in Spatial Syllogisms, 88.0% in Discrimination of Differences, 90.8% in Logical Reasoning, 91.2% in General Intelligence, and 90.6% in Intelligence Quotient (IQ) for the total sample.

**Table 3:** Logistic regression for the predictive probability of each one of the cognitive aptitudes of the BADYG-E2 regarding the potential for no repeating the course

Variable		*χ*^2^	*R*^2^	*B*	ET	Wald	*p*	OR	CI 95%
Verbal Reasoning		114.13	.38	0.43	0.52	71.18	< .001	1.54	1.39–1.71
	Constant	− 1.93			0.42	21.60	< .001	0.14	
Numerical Reasoning		132.09	.44	0.39	0.04	62.95	< .001	1.47	1.33–1.62
	Constant	− 1.57			0.36	18.59	< .001	0.20	
Spatial Reasoning		71.04	.24	0.30	0.04	54.11	< .001	1.35	1.24–1.46
	Constant	− 1.17			0.39	8.95	.03	0.30	
Verbal Syllogisms		93.99	.32	0.45	0.60	57.01	< .001	1.57	1.40–1.77
	Constant	− 1.79			0.44	16.21	< .001	0.16	
Numerical Syllogisms		63.79	.22	0.22	0.03	50.47	< .001	1.25	1.17–1.33
	Constant	− 0.82			0.37	4.98	.26	0.43	
Spatial Syllogisms		39.60	.14	0.19	0.03	35.33	< .001	1.21	1.13–1.28
	Constant	− 0.26			0.35	0.55	.45	0.76	
Attention		47.03	.16	0.26	0.41	39.46	< .001	1.29	1.19–1.40
	Constant	− 2.40			0.66	12.90	< .001	0.09	
Logical Reasoning		161.88	.52	0.21	0.25	75.29	< .001	1.23	1.17–1.29
	Constant	− 4.04			0.60	44.41	< .001	0.01	
General Intelligence		161.60	.52	0.12	0.14	73.09	< .001	1.13	1.09–1.16
	Constant	− 5.38			0.77	48.07	< .001	0.05	
Intelligence Quotient		160.98	.51	0.16	0.20	72.08	< .001	1.18	1.13–1.22
	Constant	− 12.13			1.56	59.80	< .001	0.00	

*χ*^*2*^*Chi cuadrado*, *R*^*2*^*Cuadrado de Nagelkerke*, *B Coeficiente de regresión*, *E.T. Error estándar*, *Wald Prueba de Wald*, *p Probabilidad*, *OR* odds ratio, *IC Intervalo de confianza al 95%*

Nagelkerke’s *R*^2^ (adjusted version of Cox-Snell *R*^2^, adjusting scale of the statistic to cover whole range 0 to 1) oscillated in the estimation of the adjustment value between .14 for Spatial Syllogisms and .52 for Logical Reasoning and General Intelligence. The model created for Verbal Reasoning explained 38% of the variance, Numerical Reasoning explained 44% of the variance, Spatial Reasoning explained 24% of the variance, Verbal Syllogisms explained 32% of the variance, Numerical Syllogisms explained 22% of the variance, Spatial Syllogisms explained 14% of the variance, Attention explained 16% of the variance, Logical Reasoning explained 52% of the variance, General Intelligence explained 52%, and finally, Intelligence Quotient explained 51% of the variance.

The odds ratio (OR) values were obtained for the elaborated models of the sample oscillate between 1.18 for Intellectual Coefficient and 1.57 for Verbal Syllogisms (Table 3). Thus, the probability that students do not repeat a course during the final stage of primary education is greater for each incremental point increase in the results obtained in the following indices: Verbal Reasoning 54%, Numerical Reasoning 47%, Spatial Reasoning 35%, Verbal Syllogisms 57%, Numerical Syllogisms 25%, Spatial Syllogisms 21%, Discrimination of Differences 29%, Logical Reasoning 23%, General Intelligence 13%, and in an Intelligence Quotient of 18%, for the entire sample and introducing the variables one by one.

Table 4 shows the steps followed by the model in the introduction of explanatory variables that have been significant in the probability of passing the instrumental subject of Language. For the evaluation with BADYG-E2 in the 4th year of primary education, the proposed model allows a correct estimation of 79.2% of the cases in Verbal Reasoning, 78.8% in Numerical Reasoning, 71.8% in Spatial Reasoning, 73.6% in Verbal Syllogisms, 72.4% in Numerical Syllogisms, 67.2% in Spatial Syllogisms, 67.8% in Discrimination of Differences, 83.2% in Logical Reasoning, 84% in General Intelligence, and 83.6% in Intelligence Quotient.

**Table 4:** Logistic regression for the predictive probability of each one of the cognitive aptitudes of BADYG-E2 and the student not dropping the instrumental subject of Language

Variable		*χ*^2^	*R*^2^	*B*	ET	Wald	*p*	OR	CI 95%
Verbal Reasoning		254.82	.53	0.04	0.03	121.01	< .001	1.70	1.55–1.87
	Constant	− 5.44			0.52	106.64	< .001	0.00	
Numerical Reasoning		221.04	.48	0.29	0.02	137.00	< .001	1.34	1.28–1.41
	Constant	− 3.40			0.33	106.76	< .001	0.03	
Spatial Reasoning		134.65	.31	0.29	0.03	95.48	< .001	1.34	1.26–1.43
	Constant	− 3.23			0.37	76.10	< .001	0.03	
Verbal Syllogisms		155.88	.36	0.36	0.03	99.84	< .001	1.44	1.34–1.55
	Constant	− 3.28			0.36	82.97	< .001	0.03	
Numerical Syllogisms		121.49	.29	0.23	0.02	86.80	< .001	1.26	1.20–1.32
	Constant	− 2.95			0.35	67.84	< .001	0.05	
Spatial Syllogisms		69.64	.17	0.18	0.02	58.93	< .001	1.19	1.14–1.25
	Constant	− 2.01			0.31	41.07	< .001	0.13	
Attention		78.55	.19	0.25	0.03	61.92	< .001	1.28	1.21–1.37
	Constant	− 4.19			0.57	53.09	< .001	0.01	
Logical Reasoning		325.14	.64	0.23	0.02	128.03	< .001	1.26	1.21–1.31
	Constant	− 7.96			0.73	117.92	< .001	0.00	
General Intelligence		318.02	.63	0.13	0.01	125.26	< .001	1.14	1.11–1.17
	Constant	− 9.23			0.84	118.44	< .001	0.00	
Intelligence Quotient		319.28	.63	0.18	0.01	126.47	< .001	1.20	1.16–1.23
	Constant	319.28			1.48	123.61	< .001	0.00	

*χ*^*2*^*Chi cuadrado*, *R*^*2*^*Cuadrado de Nagelkerke*, *B Coeficiente de regresión*, *ET Error estándar*, *Wald Prueba de Wald*, *p Probabilidad*, *OR* odds ratio, *IC Intervalo de confianza al 95%*

Nagelkerke’s *R*^2^ has oscillated in the estimation of the adjustment value between .17 for Spatial Syllogisms and .64 for Logical Reasoning. The model created for Verbal Reasoning accounted for 53% of the variance, for Numerical Reasoning explained 48% of the variance, for Spatial Reasoning explained 31% of the variance, for Verbal Syllogisms explained 36% of the variance, for Numerical Syllogisms explained 29% of the variance, for Spatial Syllogisms explained 17% of the variance, for Attention it accounted for 19% of the variance, for Logical Reasoning it explained 64% of the variance, for General Intelligence it explained 63%, and for Intelligence Quotient it explained 63% of the variance.

The odds ratio (OR) was obtained for the elaborated models of the sample oscillate between 1.14 for General Intelligence and 1.70 for Verbal Reasoning (Table 4). Thus, the probability that students do not drop the Language course during the final years of primary education is greater for each incremental point increase in the result obtained in the following indices: Verbal Reasoning 70%, Numerical Reasoning 34%, Spatial Reasoning 34%, Verbal Syllogisms 44%, Numerical Syllogisms 26%, Space Syllogisms 19%, Discrimination of Differences 28%, Logical Reasoning 26%, General Intelligence 14%, and Intellectual Coefficient 20%, for the whole sample and introducing the variables one by one.

In Table 5, the introduction of explanatory variables that have been significant for the probability of not dropping the instrumental subject of Mathematics can be observed. For the evaluation with BADYG-E2 in the 4th year of primary education, the proposed model allows a correct estimate for the total sample in Verbal Reasoning 76.2%, Numerical Reasoning 84.4%, Spatial Reasoning 71.6%, Verbal Syllogisms 73%, Numerical Syllogisms 73.2%, Spatial Syllogisms 71.4%, Discrimination of Differences 66.8%, Logical Reasoning 84.8%, General Intelligence 85.2%, and Intelligence Quotient 86%.

**Table 5:** Logistic regression for the predictive probability of each one of the cognitive aptitudes of the BADYG-E2 of not dropping the instrumental subject of Mathematics

Variable		*χ*^2^	*R*^2^	*B*	ET	Wald	*p*	OR	CI 95%
Verbal Reasoning		192.57	.42	0.40	0.03	111.60	< .001	1.52	1.39–1.62
	Constant	− 4.15			0.42	94.63	< .001	0.01	
Numerical Reasoning		311.81	.62	0.40	0.03	148.40	< .001	1.50	1.40–1.60
	Constant	− 4.77			0.42	125.06	< .001	0.00	
Spatial Reasoning		146.00	.34	0.31	0.03	100.39	< .001	1.37	1.28–1.45
	Constant	− 3.47			0.38	82.01	< .001	0.03	
Verbal Syllogisms		146.06	.34	0.34	0.03	96.36	< .001	1.41	1.32–1.51
	Constant	− 3.13			0.35	80.00	< .001	0.04	
Numerical Syllogisms		145.74	.33	0.26	0.02	97.35	< .001	1.30	1.23–1.37
	Constant	− 3.43			0.38	79.57	< .001	0.03	
Spatial Syllogisms		99.25	.24	0.22	0.02	76.75	< .001	1.25	1.19–1.31
	Constant	− 2.60			0.34	58.64	< .001	0.07	
Attention		76.55	.19	0.25	0.03	60.71	< .001	1.28	1.20–1.36
	Constant	− 4.15			0.57	52.47	< .001	0.01	
Logical Reasoning		367.07	.69	0.27	0.02	123.02	< .001	1.32	1.25–1.38
	Constant	− 9.47			0.88	115.62	< .001	0.00	
General Intelligence		374.67	.70	0.16	0.01	118.05	< .001	1.18	1.14–1.21
	Constant	− 11.693			1.09	113.94	< .001	0.00	
Intelligence Quotient		375.71	.70	0.22	0.02	120.13	< .001	1.25	1.20–1.30
	Constant	− 20.74			1.90	118.56	< .001	0.00	

Nagelkerke’s *R*^2^ has oscillated in the estimation of the adjustment value between .19 for Discrimination of Differences and .70 for General Intelligence and Intelligence Quotient. The model created for Verbal Reasoning accounted for 42% of the variance, for Numerical Reasoning explained 62% of the variance, for Spatial Reasoning explained 34% of the variance, for Verbal Syllogisms explained 34% of the variance, for Numerical Syllogisms explained 33% of the variance, for Spatial Syllogisms explained 24% of the variance, for Attention explained 19% of the variance, for Logical Reasoning explained 69% of the variance, for General Intelligence explained 70%, and for Intelligence Quotient explained 70% of the variance.

The odds ratio (OR) obtained for the elaborated models of the sample oscillate between 1.18 for General Intelligence and 1.50 for Verbal and Numerical Reasoning (Table 5). Thus, the probability that students do not drop the instrumental subject of Mathematics during the latter years of primary education is greater for each incremental point increase in the result obtained for the whole sample and introducing the variables one by one in the following indices: Verbal Reasoning 50%, Numerical Reasoning 50%, Spatial Reasoning by 37%, Verbal Syllogisms 41%, Numerical Syllogisms 30%, Spatial Syllogisms 25%, Discrimination of Differences 28%, Logical Reasoning 32%, General Intelligence 18%, and Intelligence Quotient 18%.

## Discussion and conclusions

Following the analysis of the obtained results and regarding the relationship between maintaining the same mean level inaptitude development in the different measured competences and the probability of repeating the course in the latter stage(s) of primary education, we can conclude that the research hypothesis (H_a_) is fulfilled, because the skills in Vocabulary and Verbal Reasoning are the variables that were measured with greater predictive capacity. Therefore, these results confirm that regarding the BADyG-E2r test which is taken at the end of primary education, it has a good capacity for predicting the appearance of difficulties in students who, in initial courses of primary education, did not present notable difficulties in overcoming the curricular objectives of the instrumental subjects. However, those students did have low scores in the aforementioned aptitudes during those initial courses. When evaluating a student’s aptitude development midway through primary education using the BADyG-E2r battery, it was detected that with each incremental point increase in the results in the skills related to Language (Vocabulary and Verbal Reasoning), the probability that the student does not repeat a subjects oscillates between 57 and 54% respectively. These results denote that, according to a student’s promotion to the next grade in their educational training, an adequate understanding and a correct usage of Language is fundamental to achieving the different curricular objectives. These data reinforce the results of previous research that also concluded that verbal aptitudes are strong predictors of learning difficulties (Bennett et al., 1997; Burnet & Lane, 1980; Cooper & Reagan, 1982; Pérez et al., 2005; Robles & Vázquez, 2014). This conclusion reinforces the importance of these skills being specifically used in student training in order to enhance student development, and therefore, such training is considered an adequate academic response, which favors a student’s acquisition of the proposed curricular objectives with respect to the different disciplines in order to correctly complete primary education.

In the same line of thought, when analyzing the results of which aptitudes have a greater predictive capacity of potentially dropping subjects at the end of primary education, we observed that in the BADyG-E2r test and for the subject of Language, the Verbal Reasoning Index has a greater predictive capacity; insofar as for each incremental point increase in the result for the Verbal Reasoning ability, the probability that the student does not drop the instrumental subject of Language increases by 70%, which in turn does not completely fulfill the research hypothesis (H_b_). Justification that knowledge of vocabulary (Verbal Syllogisms) loses importance at the predictive level of learning difficulties at the end of primary education may be due to the fact that in the latter years of primary education, a greater employment of logic that is more reflective of Language is required. Similarly, for the instrumental subject of Mathematics, we obtained a result which showed that the skills with greater predictive ability are Verbal Reasoning and Numerical Reasoning, to the extent that for each incremental point increase for the abilities in Verbal Reasoning or Numeric Reasoning, the probability that the student does not drop the instrumental subject of Mathematics increases by 52 and 50%, respectively. Therefore, our research hypothesis (H_c_) is not completely fulfilled. It may seem unusual that from the analysis of the results for the aptitudes measured in the instrumental subject of Mathematics using the BADyG-E2r test, the Verbal Reasoning Index stands out as the best predictor, over the rest of the aptitudes, of potentially dropping a course. This fact can be justified because if a student in primary education does not understand the concepts that are employed to explain the numerical operations or problems, then that student will be unable to correctly solve Mathematical exercises, due to the great level of abstraction required by cognitive processes associated with this type of activity at this educational level. In the latter years of primary education, the discipline of Mathematics requires the resolution of numerical problems to a greater extent than numerical analogies. These results are in agreement with those obtained by Harvey and Miller (2016), whose conclusions highlight the importance of the development of Mathematical Language in the early stages of education for the appropriate acquisition of Mathematics in later stages.

On the other hand, indices such as Spatial Reasoning or Spatial Syllogisms do not stand out as predictors of academic success in the instrumental subject of Language, which fulfills our hypothesis (H_d_). This contrasts with the results obtained in other studies in which these skills are understood to be determinants in the correct acquisition of reading and writing, and reading comprehension skills (Bender, 1977; Koppitz, 1980; Mlodnosky, 1968; Spitz, 2009; Valett, 1989). Perhaps, these data could present a more significant difference in relation to the other indices, if instead of making the estimate by taking into account the final stage of primary education, the results would be isolated at the academic level for the subject of Language, during the first cycle of primary education, which is a period in which the acquisition of reading and writing is apriority objective and an academic competence that requires adequate maturation at the levels of Spatial Reasoning and Spatial Syllogisms.

Another noteworthy result, which confirms our hypothesis (H_d_) and coincides with different investigations, is that General Intelligence is not the most relevant variable in terms of predicting school success at a general level or in specific academic areas such as Mathematics or Language (Deary, Strand, Smith, & Fernandes, 2007; Edel, 2003; Laidra, & Pullman, H.,& Allik, J., 2006; Miñano & Castejón, 2008; Watkins, Lei, & Canivez, 2007). On the other hand, one must account for the fact that the above statement is controversial since in like manner, it is not difficult to compare it with other investigations with General Intelligence among their results, and General Intelligence is presented as a cognitive aptitude with a greater correlation with respect to academic success (Deary et al., 2007; Gygi, Schweizer, & Grob, 2017; Kaufman, Reynolds, Liu, Kaufman, & McGrew, 2012; Roth et al., 2015; Schult & Sparfeldt, 2016). Notwithstanding, for the sample in the present study, the highest score in this index, as measured by the BADyG E2r psychometric test, has not been associated with a greater probability of repeating a course. Therefore, although General Intelligence has some influence on student success with respect to achieving the curricular objectives of the reference course, it is not the most determining factor among the measured aptitudes.

In terms of study limitations, it is noteworthy that for future investigations, the sample of both subjects and provinces in which information was collected should be expanded, and this is among the most important limitations of this study. In addition, it would be advisable to not limit the study to a single evaluation instrument, but to include different psychometric tests that allow the collection of data from other variables beyond cognitive aptitudes, in order to be able to assert in a more substantial manner what academic successes and failures can be attributed to.

Based on the obtained results and as an implication for educational practice, we can affirm that the evaluation tool used in this study, the Battery of Differential and General Skills (BADyG-E2r), can be considered effective in anticipating learning difficulties within a program of early detection that aims to implement pedagogical strategies which favor an appropriate maturity in the aptitude levels of students, and anticipating any potential academic failure. The above points should be linked to the development of models of psychopedagogical intervention (Raver et al., 2011), which aim to reduce learning difficulties in the first cycles of primary education. This is the stage in which student difficulties that go unnoticed or are not given the necessary importance will present their first signs of deficiencies in student learning, which in many cases will accumulate on each course undertaken by the student. In fact, recent research (Serpell & Esposito, 2016) underlines the importance of these types of strategies being transferred from government institutions through relevant legislation and legislation, to educational programs that are aimed at the early prevention of learning difficulties, in order to reduce the notable current rates of school failure.

As a final conclusion and in agreement with other research that collected their data from populations in Primary and Secondary Education (Duncan et al., 2007; Harvey & Miller, 2016; Serpell & Esposito, 2016), we wish to emphasize that early academic capacity is a strong predictor of later academic ability/achievement. Children with greater knowledge and understanding of letters and numerical concepts at the beginning of compulsory schooling achieve higher academic levels at later levels than their peers who are less prepared. The relevance of this research and the obtained data pertain to the Battery of Differential and General Abilities E-2r (BADyG-E2r), which is an adequate prediction tool at an early age, providing technical arguments and objectives regarding reinforcement measures at the initial schooling level, with no evident learning difficulties being present at that time.

## Data Availability

Data are available from the first author.

## Abbreviations

BADYG: Differential and General Skills Battery
*χ*^2^: Chi square
*R*^2^: *R*^2^ Nagelkerke
*B*: Coefficient
*p*: Probability
OR: Odds ratio
CI: Confidence interval
